# 

**Published:** 1879-05

**Authors:** 


Pamphlets Received: Ninth and Tenth Annual Reports of the Maryland Eye and Ear Institute. Baltimore, Maryland. 1879.

Announcement of Vassar College, for the Higher Education of Women. Poughkeepsie, New York. 1879.

The Proceedings of the Medical Society of the county of Kings. Brooklyn, New York. April, 1879.

An Address on Obstetrics and Diseases of Women and Children, delivered before the American Medical Association, June 5, 1878. By Ed. W. Jenks, M.D., Chairman of the Section of Diseases of Women and Children, etc., etc. 1878.

